# Highly phosphorylated functionalized rice starch produced by transgenic rice expressing the potato *GWD1* gene

**DOI:** 10.1038/s41598-017-03637-5

**Published:** 2017-06-13

**Authors:** Yaling Chen, Xiao Sun, Xin Zhou, Kim H. Hebelstrup, Andreas Blennow, Jinsong Bao

**Affiliations:** 1 Institute of Nuclear Agricultural Sciences, College of Agriculture and Biotechnology, Zhejiang University, Huajiachi Campus, Hangzhou, 310029 China; 2 Department of Molecular Biology and Genetics, Aarhus University, Forsøgsvej 1, 4200 Slagelse, Denmark; 3 Department of Environmental and Plant Sciences, University of Copenhagen, Thorvaldsensvej 40, 1871 Frederiksberg C, Denmark

## Abstract

Starch phosphorylation occurs naturally during starch metabolism in the plant and is catalysed by glucan water dikinases (GWD1) and phosphoglucan water dikinase/glucan water dikinase 3 (PWD/GWD3). We generated six stable individual transgenic lines by over-expressing the potato *GWD1* in rice. Transgenic rice grain starch had 9-fold higher 6-phospho (6-P) monoesters and double amounts of 3-phospho (3-P) monoesters, respectively, compared to control grain. The shape and topography of the transgenic starch granules were moderately altered including surface pores and less well defined edges. The gelatinization temperatures of both rice flour and extracted starch were significantly lower than those of the control and hence negatively correlated with the starch phosphate content. The 6-P content was positively correlated with amylose content and relatively long amylopectin chains with DP25-36, and the 3-P content was positively correlated with short chains of DP6-12. The starch pasting temperature, peak viscosity and the breakdown were lower but the setback was higher for transgenic rice flour. The 6-P content was negatively correlated with texture adhesiveness but positively correlated with the cohesiveness of rice flour gels. Our data demonstrate a way forward to employ a starch bioengineering approach for clean modification of starch, opening up completely new applications for rice starch.

## Introduction

Starch is the major carbohydrate of cereal grain and a primary dietary component of human energy intake. Grain starch also serves the major source for food and non-food applications such as in thickening, gelling, sizing, binding and adhesion. Owing to its non-optimal physicochemical properties, raw starch functionality does not meet industrial processing and product demands and therefore expensive and polluting post-harvest chemical modifications, e.g. phosphorylation producing so called “starch phosphate” to inhibit retrogradation, are frequently required^1^. Clean protocols for starch modification is therefore desired and starch bioengineering directly in the plant is an attractive approach^2^.

Phosphorylation is the only known *in vivo* covalent modification of starch. In the early 20^th^ century, the presence of small amounts (0.2–0.4% w/w) of monoesterified phosphate groups was detected in potato (*Solanum tuberosum*) tuber starch^3^. Subsequently, it has been verified that phosphorylation takes place in virtually all plant species^4, 5^. In potato tuber starch, a majority of the phosphate monoesters (70–80%) are bound at the C-6 position of the glucosyl unit, while C-3 phosphorylation makes up 20–30%^6^. Two types of glucan water dikinaseshave been demonstrated to catalyse starch phosphorylation: α-Glucan, water dikinase 1 (GWD1; formerly designated as R1 or SEX1) and α-Glucan, water dikinase 3/phosphoglucan, water dikinase (PWD/GWD3). GWD1 specifically phosphorylates starch at the C-6 position, catalyzing the transfer of the β-phosphate from ATP to a glucosyl residue^7^ and then PWD recognizes the C-6 pre-phosphorylated glucan and subsequently phosphorylates C-3 hydroxyls^8, 9^. Phosphorylation by GWD1 disrupts the crystalline structure of the starch surface and increases the hydrolytic action of plastidial β-amylases^10, 11^. RNA interference-mediated down-regulation of *GWD1* in the wheat endosperm resulted in decreased starch-bound phosphate, an increase in grain size and plant biomass but unaltered starch content^12^. Starch phosphorylation stimulates starch degradation in *Arabidopsis* mutant plants in which the activity of GWD1 or PWD/GWD3 is decreased. As a result, these plants exhibit significantly increased leaf starch contents with the phenotype of the *GWD1*-deficient plants being more severe^8, 9, 13^. However, the GWD1 enzyme level remains largely constant throughout the diurnal cycle and also starch biosynthesis seems to be affected by starch phosphorylation as demonstrated by both metabolic and structural effects in *Arabidopsis* ^14^ and barley^15^. Expression of the potato *GWD1* in barley also affects grain germination in barley^16^. In support for a role of starch phosphorylation in both biosynthesis and degradation, GWD1 catalyzed phosphorylation of crystalline starch induces physical repulsion between starch segments^5^ thereby solubilising crystalline structures^17^ providing accessibility for biosynthetic or hydrolytic enzymes. Hence, GWD1 and PWD/GWD3 play complex and dependent key roles in starch degradation and biosynthesis pathways.

From a technological point of view, mono-phosphorylated starch has increased hydration capacity, thereby influencing starch pasting properties, gel strength and clarity, stickiness and viscosity^18–20^. Hence, phopshorylated starch can improve starch functionality and can be possibility produced directly in the cereal grain, which shows tremendous potential economic and environmental advantage^2^. Engineering the expression of *GWD1* or *PWD*/*GWD3* is the obvious strategy to alter starch phosphate in the crop. The manipulation of GWD1 activity in the crop has been described in some patents including the overexpression in wheat^21^ and corn^22^ leading to increased viscosity of the starch paste. Overexpression of the potato *GWD1* gene specifically in the developing barley endosperm resulted in grain starch with ten-fold increased phosphate content, and starch granules showing altered morphology and lower melting enthalpy^15, 16, 23^.

Rice is a crucial cereal crop in developing countries and the milled grains are composed of approximately 80–90% starch^24^. Rice endosperm starch contains low concentrations of starch bound phosphate esters (≈1 nmol/mg)^3, 8^, which limits its usage in various industrial processes. Whether introduction of potato *GWD1* gene into rice could produce starch with higher bound phosphate esters needs to be addressed, which may introduce starch phosphate into the smallest granule found among cereals. The objective of this study is to express the potato *GWD1* gene in rice (*japonica*, cv Zhonghua 11) endosperm amyloplasts, and to investigate the morphological and physicochemical properties of the resulting modified rice starch.

## Results

### Production of transgenic GWD1 overexpression rice plants

To determine whether rice starch phosphate esterification could be increased by genetic modification and whether the starch functional properties could be improved to widen its application potential, we generated rice lines overexpressing the gene (*StGWD1*) coding for the potato starch phosphorylator GWD1. Stable transgenic rice lines were generated by *Agrobacterium*-mediated transformation using pUCE_D-Hord:TP-StGWD:NOS_ vector (Supplementary Fig. 1) that enables endosperm-specific overexpression of transgenic protein and targeting to the amyloplasts by an N-terminal transit peptide (TP)^25^. Sixteen transgenic lines among 24 candidate plants in the T_0_ generation were identified by genomic PCR for hygromycin phosphotransferase (*HPT*) and *StGWD1* as selection markers (Supplementary Fig. 2A). These T_0_ lines were propagated by self-pollination for four generations to create homozygous transgenic lines (Supplementary Fig. 2B). Six stable individual transgenic lines were obtained, defined as RGD1-6, respectively (Supplementary Fig. 2C).

**Figure 1:**
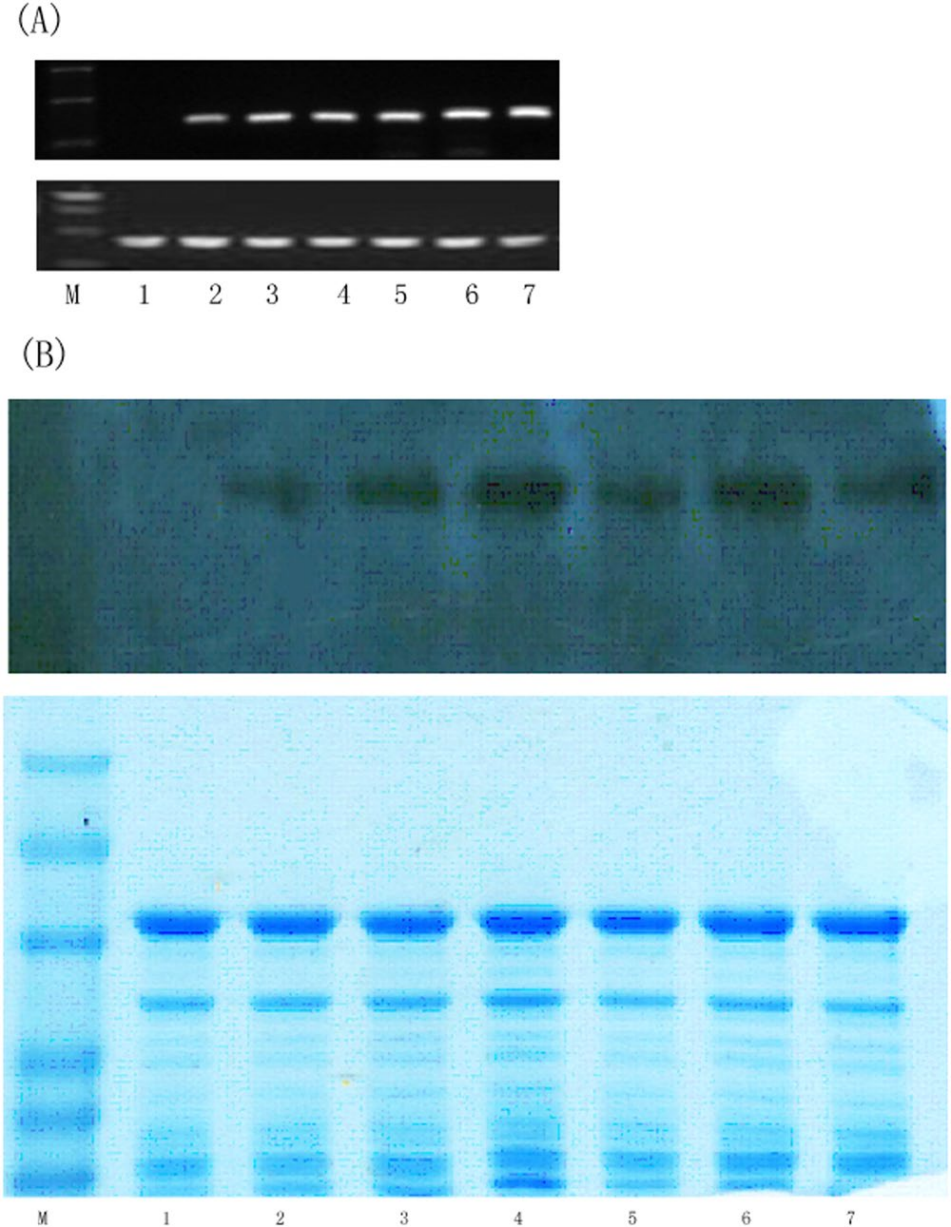
(**A**) Expression of *StGWD1* in the developing endosperm of stable offspring lines of transgenic rice (RGD1-6) at the transcript level determined by qRT-PCR (up: *StGWD1*, down: actin), and (**B**) StGWD1 protein determined by western blotting (up: StGWD1, down: the total protein). The gels were cropped to only show the relevant bands. M: marker, 1: control, 2-7: RGD1-6.

Measurements of expression of the *StGWD1* gene in developing endosperm were carried out using qRT-PCR. All six selected transgenic lines of RGD1-6 had high expression levels of *StGWD1* (Fig. 1A). The presence of StGWD1 protein in the developing endosperms was confirmed in the RGD1-6 lines by western blotting (Fig. 1B).

### Starch phosphate content

**Figure 2:**
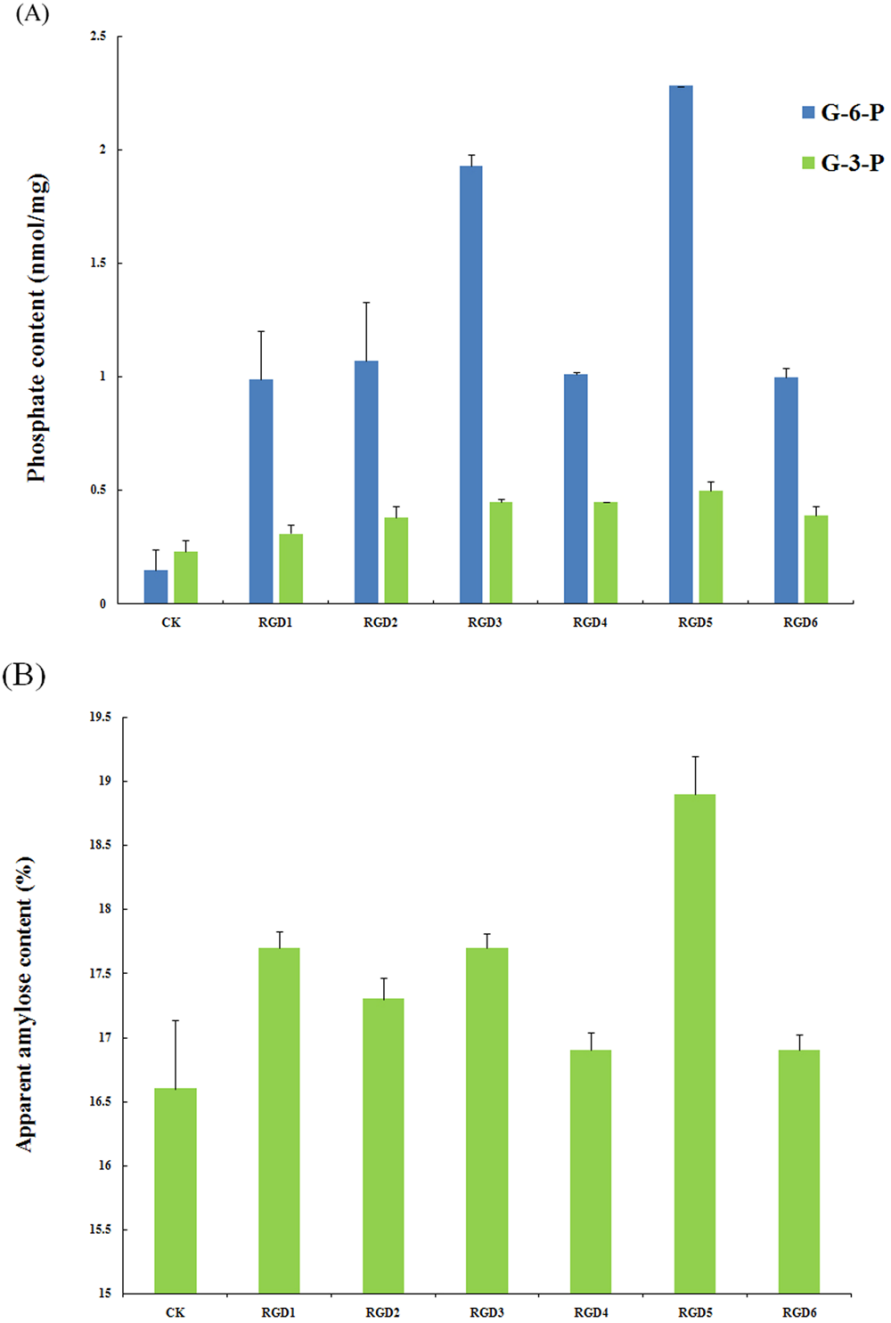
(**A**) Content of starch bound Glc-6-P and Glc-3-P (nmol mg^−1^ starch) in starch from RGD1-6 lines, and (**B**) Apparent amylose content (%) in starch from the same lines as in A.

The contents of Glc-6-P and Glc-3-P units were very low in starch of the control cultivar Zhonghua11, 0.15 nmol/mg and 0.23 nmol/mg, respectively, corresponding to that only one of approx. 30 000–50 000 glucose units in the starch is phosphomonoesterified (Fig. 2A). The transgenic rice grains had 9-fold higher Glc-6-P content as compared to control (Fig. 2A). These data are consistent with the barley system expressing the same gene^23^. Also the Glc-3-P content was double fold higher in the transgenic lines than the control. This is also consistent with the activity of PWD/GWD3 being strictly dependent on pre-phosphorylated starch^8, 9^. The concentrations of Glc-6-P and Glc-3-P varied among the transgenic lines, the RGD-5 having the highest Glc-6-P content. This variation followed the expression of the StGWD1gene.

### Starch granule morphology

**Figure 3:**
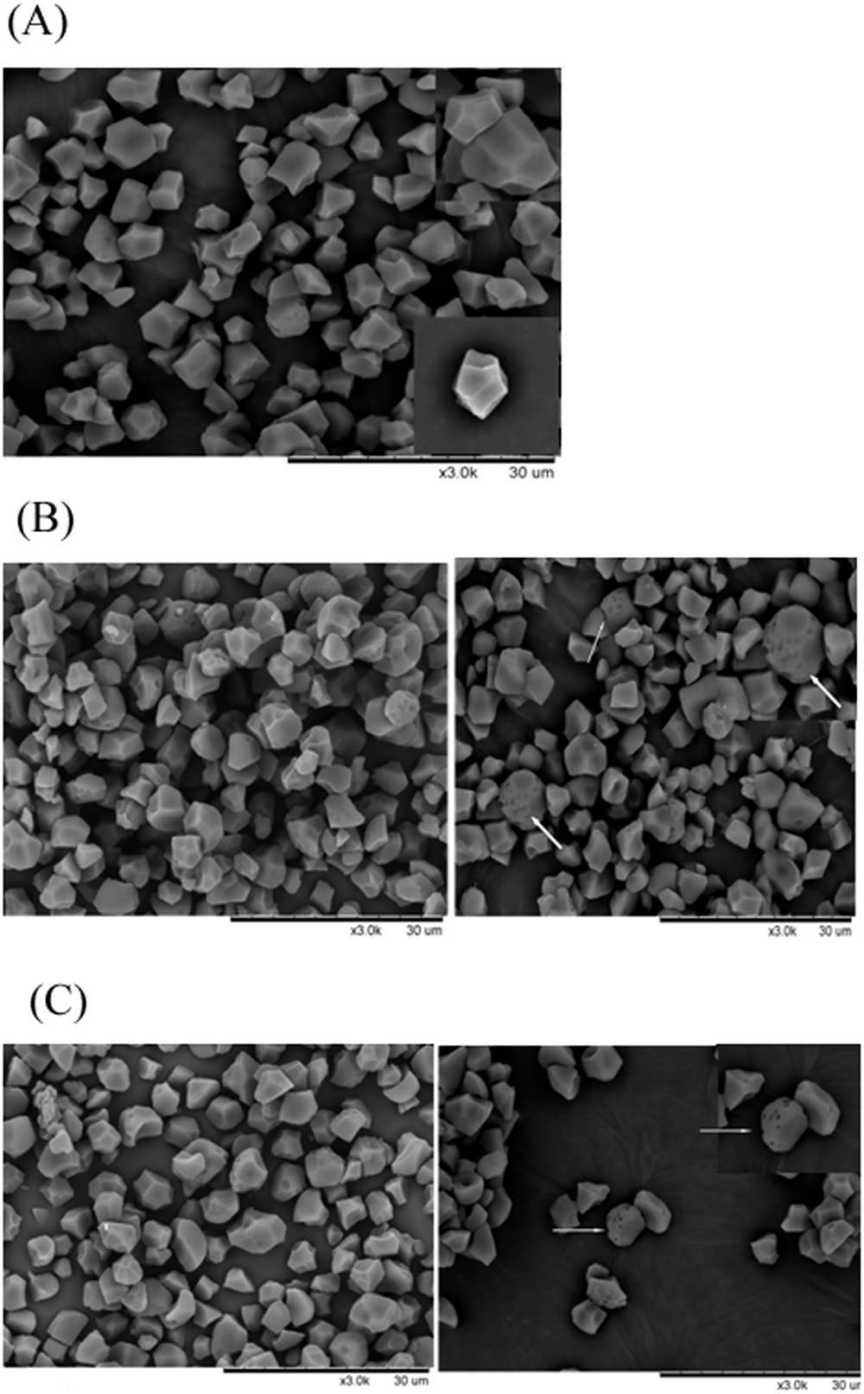
Starch granules visualized by Scanning Electron Microscopy at 3000×. (**A**) Control (Zhonghua 11). (**B**) RGD3. (**C**) RGD5. White arrows indicate pores in the starch granule. Selected starch granules in central part are magnified at 5000× and shown at the lower -right (**A**) and upper-right corners (**B** and **C**).

The transgenic starch granules had similar polygonal shapes as control Zhonghua 11 (Fig. 3). However, some granules had blurred edges and more irregular surfaces, especially granules from lines RGD3 and RGD5. Some starch granules had pores visible at the surface. A similar observation was seen on starch granules from barley plants with overexpression of *StGWD1* ^23^ demonstrating a phosphate stimulated destabilization of the starch granules as proposed^5^.

### Apparent amylose content (AAC)

The AAC was higher in all the transgenic lines as compared to control (Fig. 2B) and the Glc-6-P content was positively correlated with the AAC (r = 0.871, *P* < 0.05, Supplementary Table 2).

### Pasting viscosity

Pasting characteristics of suspensions of 3.0 g flour in 25.0 g of distilled water were analyzed by RVA, and the result showed that the peak viscosity (PV), pasting temperature (PT), breakdown (BD) and setback (SB) were significantly lower for transgenic lines as compared to control (Table 1). The Glc-6-P and Glc-3-P content were negatively correlated with PT, PV and BD, but positively correlated with SB (Supplementary Table 2).Pasting viscosity is expected to be enhanced by the presence of starch phosphomonoesters^19^. Hence, these data suggest that the pasting properties of the flour from the transgenic lines may be due to the increased starch phosphates as well as higher AAC content in these lines since amylose is known to suppress starch pasting^26^.

**Table 1:** Pasting Properties of flour from RGD1-6 by Rapid ViscoAnalyser.

	PV(RVU)	HPV (RVU)	BD (RVU)	CPV (RVU)	SB(RVU)	CS (RVU)	PT( °C)
CK	270.7a	186.4ab	84.3a	291.6a	20.9e	105.1ab	70.5a
RGD1	249.2ab	180.4abc	68.8b	287.9a	38.7d	107.5ab	68.0b
RGD2	230.9b	172.9bc	58.0bc	283.2ab	52.3bc	110.3a	65.8c
RGD3	229.7b	183.6abc	46.1c	287.4a	57.7b	103.8ab	64.9c
RGD4	251.2ab	196.3a	54.9c	300.3a	49.1c	104.1ab	67.4b
RGD5	197.1c	164.2c	32.9d	266.0b	68.9a	101.8b	64.9c
RGD6	230.7b	184.7ab	46.0c	288.7a	58.1b	104.1ab	65.8c

Rapid Visco Units (RVU), peak viscosity (PV), hot pasteviscosity (HPV), breakdownviscosity (BD), cool paste viscosity (CPV), setback viscosity(SB), consistency viscosity (CS) and pastingtemperature (PT). Different letter indicates significant difference (P < 0.05)

### Gel texture

The hardness of gels prepared from rice flour of RGD3-6 was significantly higher than that of the control. However, gels prepared from RGD1 and RGW2 flour had lower gel hardness than control (Table 2). The adhesiveness of RGD3 and RGD5 flour was significantly lower than that of the control. By contrast the cohesiveness of these lines was significantly higher (Table 2). The other transgenic lines had adhesiveness and cohesiveness similar to that of the control (Table 2). Starch phosphate is predicted to weaken the gel network^27^, while amylose content has been reported to be positively related to gel hardness^28, 29^, and the interplay between phosphate and amylose in paste and gel systems can be complex^27, 30^. For the transgenic rice lines, the higher amylose content can explain the increased hardness of GWD1 and GWD2 flour.

**Table 2:** Textural profile of the gels prepared from rice flour. Different letter indicates significant difference (P < 0.05).

	Hardness(g)	Adhesiveness(g.s)	Cohesiveness
CK	14.6cd	−22.5ab	13.5c
RGD1	13.6d	−22.5ab	12.4c
RGD2	14.1cd	−22.3ab	12.9c
RGD3	18.7b	−28.8c	17.2ab
RGD4	16.1b	−23.1ab	15.0bc
RGD5	21.2a	−31.5c	19.6a
RGD6	16.0c	−24.9b	14.3c

### Differential scanning calorimetry

The dissolution temperature parameters T_o_, T_p_ and T_c_ of transgenic starch in water were significantly lower than those of the control (Table 3). Also, the dissolution enthalpy, ΔH, of transgenic starch was significantly lower than control (Table 3). The degree of phosphorylation, i.e. the Glc-6-P and Glc-3-P content, was strongly negatively correlated with the gelatinization parameters T_o_, T_P_ and T_c_ (r = −0.871, −0.921 and −0.898, respectively) (Supplementary Table 3) while only very weak negative correlations were found for the ΔH parameter. For the barley system T_o_, T_p_ and T_c_ are not different for highly phosphorylated transgenic barley lines as compared to the control, however ΔH is significantly lower^23^. For the potato system, the low starch phosphate contents from a *GWD1* suppressor lines does not significantly affect the T_o_, T_p_ and T_c_ but ΔH is increased, indicating disturbed crystalline perfection induced by phosphate esters^5, 31^. The decrease in dissolution temperatures with starch phosphorylation found in the rice system could be directly associated with phosphate-induced destabilization of crystalline starch in the granules. Since the effects of phosphate are seemingly not the same for different plant and organ systems, additional factors can have influence on starch granule dissolution including the amount and distribution of amylose, protein and lipids.

**Table 3:** Thermal properties of flour and starch of transgenic rice as measured by DSC.

	T_o_(°C)	T_p_(°C)	T_c_(°C)	ΔH(J/g)
**Flour**				
CK	63.5a	70.2a	75.5a	7.7a
RGD1	58.8b	65.3b	71.9b	5.8c
RGD2	57.1bc	63.5cd	67.5c	7.3a
RGD3	55.9c	62.6de	67.2c	6.2bc
RGD4	57.7bc	64.6bc	70.9b	6.6b
RGD5	56.2c	61.3e	66.0c	6.0c
RGD6	58.6b	64.6c	72.2b	6.2bc
**Starch**				
CK	63.7a	69.3a	75.2a	12.0a
RGD1	58.5b	63.3b	66.3bc	11.6ab
RGD2	57.2c	62.1c	67.3b	11.8ab
RGD3	55.9d	61.1d	65.3bc	10.2c
RGD4	57.6c	62.7bc	66.3bc	11.1abc
RGD5	55.7d	60.4d	64.6c	10.5bc
RGD6	57.9bc	62.7bc	65.6c	10.9abc

Different letter indicates significant difference (P < 0.05).

### Chain length distribution

Surveying starch samples cross species, relations are found for the chain length and crystalline polymorphs of the starch granules^4^. Typically, phosphorylated starches have longer amylopectin chains than non-phosphorylated ones and highly phosphorylated starches specifically shows an increasing proportion of chains with at DP 19^4^. To investigate if there is a similar effect in rice, the amylopectin chain length distribution was analyzed by HPAEC-PAD (Table 4). The unit chains of debranched amylopectin can be grouped into fa (DP 6-12), fb_1_ (DP 13-24), fb_2_ (DP 25-36) and fb_3_ (DP ≥ 37) populations^32^. The profiles of the control and transgenic lines were rather similar but specific changes could be detected. Generally, the average chain length of amylopectin from transgenic lines was higher, which is in agreement with the notion that phosphorylated chains in starch granule are typically longer, compared to non-phosphorylated amylopectin chains^4^. Compared to control, the short main peak DPs of transgenic lines were somewhat decreased (from DP15 to DP14) and the content of longer chains were increased in lines RGD1, RGD2 and RGD5. Hence, we found fewer chains in the fb_1_ (DP 13-24) population and more chains in the fb_3_ (DP ≥ 37) population as compared to control. Correlation analysis demonstrated that the Glc-6-P content was negatively correlated with the proportion of DP 13-14 (r = −0.863), but positively correlated with the proportion of DP 25-36 (r = 0.941) (Supplementary Table 3). The Glc-3-P content was positively correlated with the proportion of DP 6-12 (r = 0.840) (Supplementary Table 3). However, for the barley system expressing the *StGWD1* gene, no significant relationship was found for amylopectin chain length and starch phosphorylation^15^ demonstrating the increasing the phosphorylation by overexpressing the *GWD1* gene does not significantly affect the amylopectin chain structure.

**Table 4:** Chain-length distributions of amylopectin. Different letter indicates significant difference (*P* < 0.05).

Sample	Peak dp		Average CL	% Distribution			
	I	II		dp6-12	dp13-24	dp25-36	dp ≥ 37
CK	15	38	33.2	19.87a	50.91a	16.43a	12.78a
RGD1	14	42	35.1	19.56a	47.53a	16.85a	16.06a
RGD2	14	43	34.5	20.22a	48.12a	16.70a	14.97a
RGD3	14	38	34.6	20.87a	46.70a	17.39a	15.04a
RGD4	14	38	33.7	20.72a	49.20a	16.59a	13.48a
RGD5	14	40	34.4	20.88a	47.00a	17.43a	14.68a
RGD6	14	38	33.4	20.87a	49.67a	16.59a	12.87a

## Discussion

Potato glucan water dikinase 1 (StGWD1) is a 155 kDa protein, and is known to be involved in starch metabolism by adding phosphate groups to amylopectin^33, 34^. Homologs of StGWD1 have been found in many other plants and organs such as tubers, endosperms of cereals, fruits and leaves, demonstrating the high conservation of GWD1^35, 36^. Several studies were made to alter the expression of GWD1 homologs in potato^19, 31^, barley^23^, maize^37^, and wheat^12^, and mutant analyses aimed at economically important traits, such as physicochemical properties of starch, starch content, and plant biomass. The endosperm-specific inhibition of GWD1 homologs in wheat by RNAi decreases the amount of grain starch-bound Glc-6-P by up 70%, and increases plant biomass^12^. Carciofi *et al*. reported about a 10-fold increase in grain starch-bound phosphate in barley by over-expressing of *StGWD1* in endosperm amyloplasts, and the high starch phosphate content in these transgenic lines results in altered granule morphology and affects the physicochemical properties of starch^23^. In this study, we generated six stable individual transgenic lines over-expressing of *StGWD1* in rice (*japonica*, cv. Zhonghua 11) endosperm, which lead to about 9-fold and double increase in Glc-6-P and Glc-3-P, respectively, consisting with the results of Carciofi *et al*.^23^. The degree of substitution for industrial, chemically modified monostarch phosphate is less than 0.5% (FAO, http://www.fao.org/docrep/w6355e/w6355e0o.htm) corresponding to approximately 50 nmol/mg. This level is in the same range as for highly phosphorylated potato starch^5^. For the chemically modified rice starch, the phosphate content was increased by 3-fold or 4-fold, which displayed significantly increased food freeze-thaw stability^38^. Although the phosphate content in the transgenic rice was not as high as highly phosphorylated potato, we suppose that the content of starch phosphate in these lines is in a concentration range sufficient for application where stabilized starch is required.

The starch granules of transgenic lines showed only minor morphologically differences including the presence of surface pores and slightly distorted edges and irregular surfaces found in some granules. Molecular force-field models indicate that a phosphate ester can be linked at the C-6 position to amylopectin without disturbing its double helical structure^39, 40^. The same models show that a phosphate ester at the C-3 position imparts molecular strain in the double helical motif thereby preventing optimal crystalline packing^39, 40^. Following studies indicated that starch phosphate esters can stimulate hydrolytic enzyme activity *in vitro* and have a stabilizing effect on the β-amylase-glucan ligand complex, supporting that starch phosphate esters can direct amylases to phosphorylated spots by GWD1 and PWD/GWD3 catalyzed on starch granules^10, 41^. Hence, the presence of pores in starches with *GWD1* over-expressed rice lines indicates that the presence of GWD1 seems to stimulate starch degradation in rice endosperm.

GWD1 efficiently catalyzes the phosphorylation of crystalline maltodextrins and thereby induces the solubilization of both the neutral and the phosphorylated glucans in these crystalline aggregates^17^. Phosphorylation also influences the physicochemical properties of starch, i.e. decreases the melting enthalpy for starch as deduced from starch extracted from tubers of *GWD1* antisense suppressed potato lines^31^. DSC data for the transgenic rice starch showed that the dissolution temperature parameters T_o_, T_p_ and T_c_ and dissolution enthalpy (ΔH) of hyper-phosphorylated starch in *GWD1* over-expression lines were significantly lower than control. Amylose is known to suppress ΔH^31^, so the suppression of ΔH can be a combined effect of increased amylose and starch phosphate. The low dissolution temperatures found can be an effect of phosphate-induced disturbance of the crystalline lattice of the granules even though this parameter was not affected in the potato system^31^. In the barley system T_o_, T_p_, T_c_ are not different for hyper-phosphorylated starch as compared to control but ΔH is decreased despite the constant amylose content^23^. Our correlation analysis for the rice system demonstrated that the degree of phosphorylation in *GWD1* over-expressed rice lines was negative correlated with ΔH, but not at significant level. For potato tuber starch, a strong negative correlation has been found between melting enthalpy of the lintnerised starch and the phosphate content^42^. Hence, the effects of starch phosphate monoesters are apparently complex not the same for different starch granule systems. Factors including the amount and distribution of amylose, protein and lipids are also important.

The AAC was higher in all the transgenic lines as compared to control (Fig. 2B) and was positively correlated with Glc-6-P content (r = 0.871, *P* < 0.05, Supplementary Table 2). This is not in agreement with data for the barley system^23^, where amylose was not affected demonstrating different metabolic routes in these two plants. For the potato tuber system, decreased starch phosphate content, due to RNA interference suppression of the *GWD1*, resulted in increased amylose content^19^. Hence, the different effects on amylose content in the phosphorylation-modified rice, barley and potato models are not consistent and needs further investigation, e.g. quantification of the amylose synthesising enzyme such as granule bound starch synthase. Pasting profiles using RVA can reveal effects of specific molecular structures, including phosphate monoesters and amylose, on starch granule swelling and disintegration of starch granules during heating and shear^43^. Especially the degree of phosphorylation is known to enhance the pasting capability of starch^18, 19^. It is reported that the pasting viscosity parameters are highly correlated with the AAC for rice starches^26, 44^. Our data showed that the AAC was negatively correlated with PV and CPV, while both G-6-P and G-3-P had significant negative correlation with most RVA parameters, notably PV and BD, but positively affected the SB and the gel hardness (Supplementary Table 2). The latter effects may be due to the combined high amylose and dynamics of phosphate-stimulating chain re-association to efficiently form an entangled gel network. Provided that the starch gelatinizes well, higher amylose content and longer amylopectin chains tend to exhibit harder gels^29^. The general negative effect on pasting behavior (except for the SB) found in the rice system is supposedly directed mainly by the high amylose content and less by the increased phosphate.

Phosphorylated starches are of tremendous value for technical applications, and this type of modification yields a significant hydration capacity producing clear and highly viscous starch pastes^19, 45, 46^. If phosphorylated starch modification carried out directly in the crop by transgenic biotechnology, modern breeding or mutagenesis, starch functionality can be improved with little or no requirement for post-harvest processing, which has tremendous potential economic and environmental advantages^2^. We here produced hyper-phosphorylated starch in rice by endosperm-specific transgenic overexpression of potato *StGWD1* resulting in an increase of starch phosphate content and the improvement of starch functionality. Our data demonstrate a way forward to employ a starch bioengineering approach for clean modification of starch.

## Conclusion

Rice starch contains only low concentrations of starch bound phosphate monoesters, which limits its usage in various industrial processes. We produced six stable individual transgenic lines with hyper-phosphorylated starch by the overexpression of the *StGWD1* in rice (*japonica*, cv Zhonghua 11). The transgenic lines had 9-fold and double higher Glc-6-P and Glc-3-P, respectively and increased amylose content. The starch granules displayed only minor morphological alterations, notably the presence of surface pores and moderately distorted edges and surfaces. The novel starch introduces unique combinations of functionality for rice starch, such as reduced gelatinization temperature, decreased pasting viscosity, increased gel formation capacity and increased gel hardness.

## Methods

### Plant materials

Rice *japonica* variety “Zhonghua11” (*O. sativa* L.) was used as the parent cultivar. The plants were grown in the greenhouse at the Zhejiang University, Huajiachi Campus, Hangzhou, China. The developing seeds were harvested 15 days after flowering (DAF), immediately frozen in liquid nitrogen and stored at −80 °C. Mature seeds were harvested 40 DAF for extraction of starch and analysis of physicochemical properties.

### Transformation

The plasmid vector pUCE_D-Hord:TP-StGWD:NOS_ was the same as that for the transformation of barley (Supplementary Fig. 1)^23, 25^. D-hordein promoters have earlier been demonstrated to direct strong endosperm specific gene expression^47^. Transformation of rice was performed according to the method of Duan *et al*.^48^.

### DNA and RNA extraction, and PCR amplification

Total genomic DNA was extracted from fresh leaves using modified cetyltrimethylammonium bromide (CTAB) method^49^. Putative transgenic plants were identified for the presence of the transgene by PCR amplification. Specific PCR primers were designed using the Primer Premier 5.0 software, according to the *StGWD1* gene and HPT gene sequence (Supplementary Table 1). The PCR reaction was performed in a volume of 10 μL containing 5 μL 2× PCR Mix buffer (ToYoBo), 50 ng genomic DNA samples and 1 μL forward and reverse primers (10 μM). PCR amplification procedure was 4 min at 94 °C followed by 35 cycles of 1 min at 94 °C, 1 min at 57 °C and 30 s at 72 °C. The PCR products were checked for the presence of fragment by agarose gel electrophoresis.

Total RNA was extracted using UNIQ-10 Column Total RNA Purification Kit (Sangon Biotech) and then reverse transcribed to cDNA using Frist-Strand Synthesis of cDNA kit (Promega) according to the manufacturer’s instructions. The specific primers used in the subsequent RT-PCR are listed in Supplementary Table 1. Actin was used as internal housekeeping control for normalization of mRNA content. The conditions and procedures for RT-PCR was the same as for standard PCR described above.

### Western Blotting

Soluble proteins were extracted according to Chen and Bao^50^, their concentration measured using NANODROP 2000 spectrophotometer (Thermo, Canada) and sodiumdodecyl sulphate polyacrylamide electrophoresis (SDS-PAGE)^51^. Separated proteins were blotted onto polyvinylidene fluoride fluoride (PVDF) membranes using a transblotter and GWD1 was detected using western blotting according to Crofts *et al*.^52^ using rabbit polyclonal antiserum directed against StGWD1 as probe^20^.

### Preparation of rice flour and starch

The mature rice grains were sun-dried until the seed moisture was around 12%, and stored at room temperature for three months^28^. Rice flour preparation was according to Bao *et al*.^28^ and starch was extracted according to Kong *et al*.^26^.

### Scanning electron microscopy (SEM)

The starch samples were attached to specimen stub and sputter coated with gold using Eiko IB5 before viewing with scanning electron microscope (TM-1000).

### Starch phosphate content and apparent amylose content (AAC)

G-6-P and G-3-P contents were quantified using high-performanceanion-exchange chromatography (HPAEC) with pulsed amperometric detection (PAD)^53^. AAC was measured using the iodine staining method^28^, calculated using a standard curve made from 4 rice samples with known amylose content.

### RVA pasting properties

The paste property of rice flour was determined byusing a Rapid Visco Analyzer (RVA-3, Newport Scientific, Warriewood, Australia). According to the method of AACC61-02, 3.0 g of rice flour was placed an aluminum canister, and 25.0 g ofdistilled water was added. The heating profile was: (1) constant 50 °C for 1 min; (2) linear ramp to 95 °C until 4.8 min; (3) constant 95 °C until 7.3 min; (4) linear ramp down to 50 °C at 11.1 min and (5) constant 50 °C until 12.5 min. Three primary parameters were calculated from the pasting curves: peak viscosity (PV), hot paste viscosity (HPV), and cool paste viscosity (CPV). Three secondary parameters were calculated from primary parameters: breakdown viscosity (BD = PV − HPV), setback viscosity (SB = CPV − PV) and consistency viscosity (CS = CPV − HPV). The viscosity parameters were measured in Rapid Visco Units (RVU). Pasting temperature (PT) was determined according to the method proposed by Bao *et al*.^28^.

### Gel texture

The sample cans containing rice starch gels formed in RVA analysis were sealed by Parafilm and kept at 4 °C for 24 h. Textural properties were determined by a TA-XT2i Texture Analyzer (Texture Technologies Corp., Scarsdale, NY) equipped with Texture Expert software (version 1.2) in a two-cycle programme. Hardness, adhesiveness, and cohesiveness were calculated using the Texture Expert software program (Version 5.16).

### Thermal properties

Thermal properties were monitored using a Differential Scanning Calorimeter model Q20 (TA Instruments, New Castle, DE, USA), and the parameters of Onset (T_o_), Peak (T_p_), Conclusion temperatures (T_c_) and enthalpy (ΔH_g_) of gelatinization were calculated by a Universal Analysis Program, version 4.4 A (TA instruments, Newcastle, DE, USA).

### Chain-Length Distribution Determination

The distribution of the amylopectin side chains was performed following enzymatic debranching using isoamylase and separation of the generated linear fragments using HPAEC-PAD^54^.

### Statistical analysis

Data were subjected to one-way analysis of variance (ANOVA) and Tukey’s test to determine significant differences by SAS statistical software (version 9.3, SAS Institute Inc., Cary, NC). Correlation analyses among the different parameters were calculated by the Proc Corr procedure.

## Electronic supplementary material

Supplementary Table 1 to 3, Fig. 1 & 2
