# Supplementary material for: Highly phosphorylated functionalized rice starch produced by transgenic rice expressing the potato GWD1 gene
Source: Sci Rep. 2017 Jun 13;7:3339. doi: 10.1038/s41598-017-03637-5 (PMC5469863; doi:10.1038/s41598-017-03637-5)
Supplement: Supplementary file 1 — Supplementary Table 1 to 3, Fig. 1 & 2 [file 41598_2017_3637_MOESM1_ESM.pdf]

Supplementary Data

Highly phosphorylated functionalized rice starch produced by transgenic rice expressing the potato *GWD1* gene

Yaling Chen[1#], Xiao Sun[1#], Xin Zhou[1], Kim H. Hebelstrup[2], Andreas Blennow[3*], Jinsong Bao[1*]

[1]Institute of Nuclear Agricultural Sciences, College of Agriculture and Biotechnology, Zhejiang University, Huajiachi Campus, Hangzhou, 310029, China.

[2]Department of Molecular Biology and Genetics, Aarhus University, Forsøgsvej 1, 4200 Slagelse, Denmark

[3]Department of Environmental and Plant Sciences, University of Copenhagen, Thorvaldsensvej 40, 1871 Frederiksberg C, Denmark

#These authors contributed equally to this work.

*Corresponding authors: jsbao@zju.edu.cn (J. B.) and abl@plen.ku.dk (A. B.).

Supplementary Table1. Primers used for the molecular detection

Primers		Sequences
HPT	[F]	5’-ATGTTGGCGACCTCGTATTT-3’
	[R]	5’-CGTTATGTTTATCGGCACTTT-3’
StGWD A	[F]	5’-AAACCAAACAACCGTCTA-3’
	[R]	5’-GATAATCTGCCAACTTCC-3’
StGWD B	[F]	5'-AGTCACCCTCAATACCGT-3'
	[R]	5'-CATAAAGCCTTCTCCCTC-3'
Actin	[F]	5'-GTCTGCGATAATGGAACTG-3'
	[R]	5'-TCTGGGTCATCTTCTCACGA-3'

Supplementary Table 2. Correlation analysis of physicochemical properties of rice flours

	G-6-P	G-3-P	AAC	To	Tp	Tc	ΔHg
G-3-P	0.869*	1					
AAC	0.871*	0.604	1				
protein	0.537	0.622	0.381				
To	**-0.871***	**-0.899****	-0.637	1			
Tp	**-0.921****	**-0.907****	-0.745	0.978***	1		
Tc	**-0.898****	**-0.825***	-0.762*	0.913**	0.935**	1	
ΔHg	-0.651	-0.536	-0.611	0.604	0.627	0.380	1
PV	**-0.896****	**-0.800***	-0.829*	0.786*	0.896**	0.855	0.532
HPV	-0.548	-0.285	-0.694	0.256	0.439	0359	0.492
BD	**-0.890****	**-0.926****	-0.716	0.924**	0.963***	0.957***	0.432
CPV	-0.670	-0.399	-0.818*	0.345	0.527	0.514	0.435
SB	**0.880****	**0.919****	0.68	-0.935**	-0.975***	-0.923**	-0.497
CS	-0.494	-0.446	-0.514	0.351	0.361	0.607	-0.174
PT	**-0.865***	**-0.856***	-0.611	0.927**	0.952***	0.881**	0.549
Hardness	**0.844***	**0.781***	0.701	-0.56	-0.645	-0.646	-0.416
Adhesiveness	**-0.892****	-0.719	-0.793	0.583	0.68	0.677	0.520
Cohesiveness	**0.841***	**0.774***	0.714	-0.551	-0.631	-0.118	-0.409

*, **, *** Indicate significance at P<0.05, 0.01, and 0.001 levels, respectively. The analysis of correlation was based on the six transgenic lines and control in the study.

Continue to Supplementary Table 2

	PV	HPV	BD	CPV	SB	CS	PT	Hardness	Adhesiveness
G-3-P									
AAC									
To									
Tp									
Tc									
ΔHg									
PV	1								
HPV	0.752	1							
BD	0.916**	0.423	1						
CPV	0.829*	0.963***	0.551	1					
SB	-0.927**	-0.468	-0.988***	-0.557	1				
CS	0.342	-0.062	0.507	0.208	-0.367	1			
PT	0.889**	0.483	0.927**	0.529	-0.963***	0.21	1		
Hardness	-0.763	-0.542	-0.718	-0.676	0.677	-0.542	-0.633	1	
Adhesiveness	0.814*	0.671	0.71	0.78*	-0.683	0.458	0.682	-0.967***	1
Cohesiveness	-0.736	-0.504	-0.704	-0.653	0.653	-0.591	-0.598	0.997***	-0.959***

*, **, *** Indicate significance at $P<0.05$, 0.01, and 0.001 levels, respectively. The analysis of correlation was based on the six transgenic lines and control in the study.

Supplementary Table 3. Correlation analysis of physicochemical properties of rice starches

	G-6-P	G-3-P	AAC	dp6-12	dp13-24	dp25-36	dp≥37	T_o	T_p	T_c
G-3-P	**0.869***	1								
AAC	**0.871***	0.604	1							
dp6-12	0.637	**0.84***	0.257	1						
dp13-24	**-0.863***	-0.628	-0.827*	-0.233	1					
dp25-36	**0.941****	0.672	0.886**	0.439	-0.887**	1				
dp≥37	0.498	0.192	0.626	-0.293	-0.853*	0.584	1			
T_o	**-0.871***	**-0.899****	-0.647	-0.629	0.82	-0.704	-0.514	1		
T_p	**-0.868***	**-0.889****	-0.666	-0.614	0.814	-0.696	-0.516	0.997**	1	
T_c	**-0.784***	**-0.828***	-0.591	-0.564	0.746	-0.605	-0.483	0.953***	0.967***	1
ΔH_g	**-0.849***	**-0.827***	-0.550	-0.831*	0.597	-0.781*	-0.123	0.732	0.721	0.709

*, **, *** Indicate significance at $P<0.05$, 0.01, and 0.001 levels, respectively. The analysis of correlation was based on the six transgenic lines and control in the study.

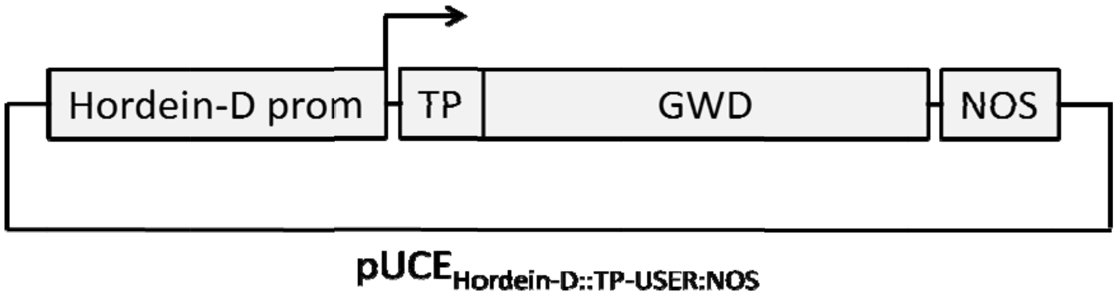

Supplementary Fig. 1. Glucan water dikinase (GWD) was cloned into the USER site of the vector pUCEHordein-D::TP-USER:NOS (Hebelstrup *et al.* 2010) as described in Carciofi *et al.* 2011. TP: Transit peptide from barley Granule-Bound Starch Synthase Ia (GBSSIa). Hordein-D prom: Promotor from barley Hordein D.

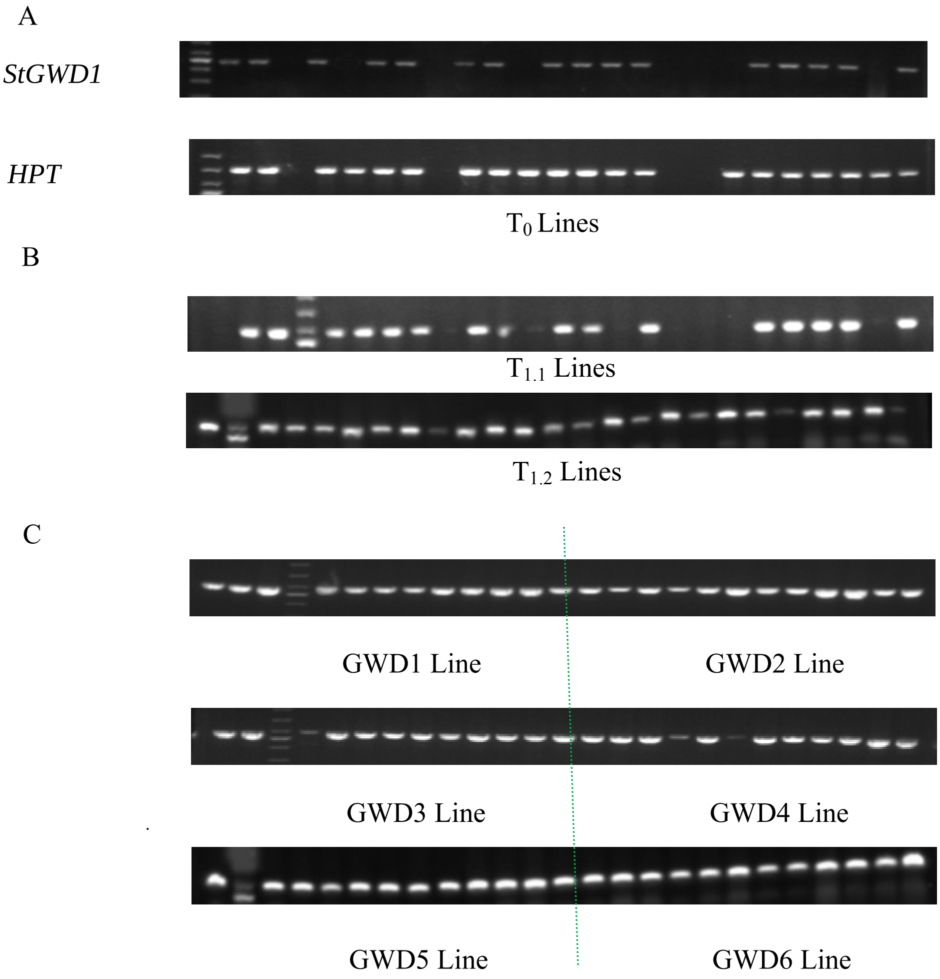

Supplementary Fig. 2 Identification of the *StGWD1* replication determined by PCR of genome. (A) Results for total of the 24 transgenic candidates (T0 generation), (B) Partial results for the first generation (T1); (C) Six stable offspring lines at T4 (12 plants/line).
